# tabAnti-HER2 (*erb*B-2) oncogene effects of phenolic compounds directly isolated from commercial Extra-Virgin Olive Oil (EVOO)

**DOI:** 10.1186/1471-2407-8-377

**Published:** 2008-12-18

**Authors:** Javier A Menendez, Alejandro Vazquez-Martin, Rocio Garcia-Villalba, Alegria Carrasco-Pancorbo, Cristina Oliveras-Ferraros, Alberto Fernandez-Gutierrez, Antonio Segura-Carretero

**Affiliations:** 1 Catalan Institute of Oncology (ICO)-Health Services Division of Catalonia, Catalonia, Spain; 2 Girona Biomedical Research Institute (IdIBGi), Girona, Catalonia, Spain; 3 Medical Oncology, Dr. Josep Trueta University Hospital of Girona, Girona, Catalonia, Spain; 4 Department of Analytical Chemistry, Faculty of Sciences, University of Granada, Granada, Spain

## Abstract

**Background:**

The effects of the olive oil-rich Mediterranean diet on breast cancer risk might be underestimated when HER2 (*ERB*B2) oncogene-positive and HER2-negative breast carcinomas are considered together. We here investigated the anti-HER2 effects of phenolic fractions directly extracted from Extra Virgin Olive Oil (EVOO) in cultured human breast cancer cell lines.

**Methods:**

Solid phase extraction followed by semi-preparative high-performance liquid chromatography (HPLC) was used to isolate phenolic fractions from commercial EVOO. Analytical capillary electrophoresis coupled to mass spectrometry was performed to check for the composition and to confirm the identity of the isolated fractions. EVOO polyphenolic fractions were tested on their tumoricidal ability against HER2-negative and HER2-positive breast cancer *in vitro *models using MTT, crystal violet staining, and Cell Death ELISA assays. The effects of EVOO polyphenolic fractions on the expression and activation status of HER2 oncoprotein were evaluated using HER2-specific ELISAs and immunoblotting procedures, respectively.

**Results:**

Among the fractions mainly containing the *single phenols *hydroxytyrosol and tyrosol, the *polyphenol acid *elenolic acid, the *lignans *(+)-pinoresinol and 1-(+)-acetoxypinoresinol, and the *secoiridoids *deacetoxy oleuropein aglycone, ligstroside aglycone, and oleuropein aglycone, all the major EVOO polyphenols (*i.e. *secoiridoids and lignans) were found to induce strong tumoricidal effects within a micromolar range by selectively triggering high levels of apoptotic cell death in HER2-overexpressors. Small interfering RNA-induced depletion of HER2 protein and lapatinib-induced blockade of HER2 tyrosine kinase activity both significantly prevented EVOO polyphenols-induced cytotoxicity. EVOO polyphenols drastically depleted HER2 protein and reduced HER2 tyrosine autophosphorylation in a dose- and time-dependent manner. EVOO polyphenols-induced HER2 downregulation occurred regardless the molecular mechanism contributing to HER2 overexpression (*i.e*. naturally by gene amplification and ectopically driven by a viral promoter). Pre-treatment with the proteasome inhibitor MG132 prevented EVOO polyphenols-induced HER2 depletion.

**Conclusion:**

The ability of EVOO-derived polyphenols to inhibit HER2 activity by promoting the proteasomal degradation of the HER2 protein itself, together with the fact that humans have safely been ingesting secoiridoids and lignans as long as they have been consuming olives and OO, support the notion that the stereochemistry of these phytochemicals might provide an excellent and safe platform for the design of new HER2-targeting agents.

## Background

Case-control, cohort, and prospective epidemiological studies have generated conflicting results regarding a protective effect of an olive oil (OO)-rich Mediterranean diet against several malignancies, especially breast cancer [1-5]. It has been assumed that, if protective agents (*e.g. *monounsaturated fatty acids such as oleic acid and/or antioxidants from olive oil and raw vegetables) are largely present in the diet of a population with a low risk of acquiring solid tumors (*i.e. *dietary patterns found in olive-growing areas of the Mediterranean basin), these diet-based anti-cancer mechanisms should influence the occurrence of all or most types of cancer. Sant *et al. *[6], by analyzing the data of the ORDET prospective study on hormones, diet and breast cancer [7], have recently suggested that the effects of the Mediterranean diet on breast cancer risk might be underestimated when HER2 (*ERB*B2) oncogene-positive and HER2-negative breast carcinomas are considered together. The Type I receptor tyrosine kinase (RTKs) HER2 regulates biological functions as diverse as cellular proliferation, transformation, differentiation, motility and apoptosis. HER2 is therefore one of the most commonly analyzed proto-oncogenes in human cancer studies, as it plays a pivotal role in oncogenic transformation, tumorigenesis, and metastasis [8-12]. *HER2 *gene is amplified and/or overexpressed in ~20% to 30% of invasive breast carcinomas and is associated with unfavorable prognosis, shorter relapse time, and decreased overall survival [8-12].

Although one observational study is not sufficient and consistency of findings across multiple cohort and case-control studies is paramount to definitely establish whether the protective effect of a diet rich in raw vegetables and OO is largely restricted to HER2-positive breast carcinomas, when considering experimental and epidemiological evidence together it is reasonable to suggest that dietary factors influencing the occurrence of HER2-positive breast carcinomas may differ from those influencing the occurrence of HER2-negative cancers [6]. Moreover, an OO, salad and vegetable-rich dietary pattern might specifically exert a protective effect against HER2-positive breast cancer because the (anti-cancer) mechanism of action of some of its components largely depends on their ability to suppress HER2 expression. We previously demonstrated that an experimental diet with a high content in Extra Virgin OO (*i.e. *the juice of the olive obtained solely by pressing and consumed without any further refining process) acted as a negative modulator on the promotion stage of dimethylbenz(a)anthracene (DMBA)-induced mammary tumors in rats by conferring to the tumors a more benign clinical behavior and lower histopathological malignancy [13]. Mechanistically, both *in vivo *and *in vitro *studies revealed that oleic acid (OA; 18:1n-9) – the main EVOO's monounsaturated fatty acid (MUFA) – transcriptionally suppressed the expression of HER2 gene [14-19]. We recently described that the polyphenol oleuropein aglycone, a non-glyceridic constituent of EVOO, was capable to reverse breast cancer acquired autoresistance to the anti-HER2 monoclonal antibody trastuzumab (Herceptin™) [20]. However, the HER2-related anti-breast cancer activities of polyphenolic compounds present in the soluble fraction of EVOO other than oleuropein aglycone, which have been suggested to contribute the oxidative stability of EVOO, and as such are often associated with the health benefits of EVOO [21-24], remained to be fully evaluated.

**Figure 1:**
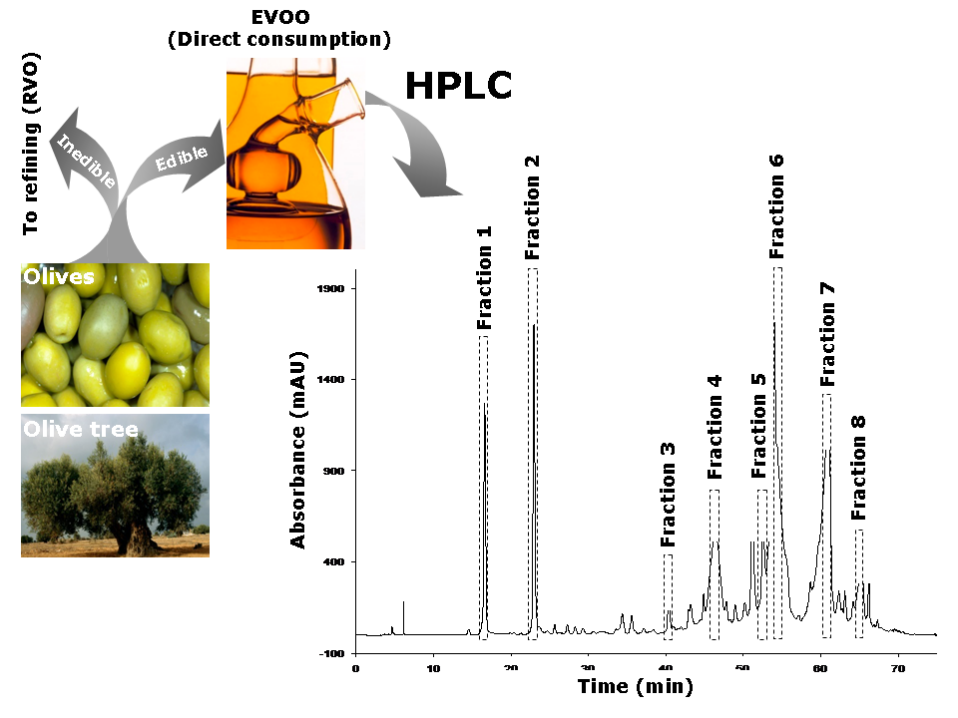
**Chromatogram of EVOO sample using semi-preparative HPLC**. Solid-liquid extraction of polyphenols from EVOO (*i.e. *the juice of the olive obtained solely by pressing and consumed without any further refining process) was performed as described in "Materials and methods". Detection was performed at 280 nm. Isolated fractions and main peak identified in each fraction: 1, hydroxytyrosol; 2, tyrosol; 3, elenolic acid (was detected at 240 nm); 4, DAOA; 5, (+)-pinoresinol;6, 1-(+)-acetoxypinoresinol;7, oleuropein aglycone;8, ligstroside aglycone.

We here designed a systematic approach to investigate the effects of EVOO-derived polyphenol compounds on the expression and oncogenic activity of the HER2 tyrosine kinase in human breast cancer-derived cell lines. EVOO is unique among other vegetable oils because of its high level of phenolic compounds [25-29]. These levels are possible because it is obtained from the olive fruit (*Olea Europea *L.) solely by mechanical means, without further treatment other than washing, filtration, decantation, or centrifugation (Figure 1). To evaluate a potential therapeutic role for EVOO polyphenols it is important to either synthesize or isolate individual compounds. Synthesis is currently not practical and, therefore, isolation procedures must be used. First, solid phase extraction followed by semi-preparative high-performance liquid chromatography (HPLC) was used to isolate phenolic fractions from commercial EVOO. Second, analytical capillary electrophoresis coupled to mass spectrometry (CE-MS) was performed to check for the composition of the isolated phenolic fractions and to confirm their identity. Third, the effects of EVOO phenolic fractions on breast cancer cell viability, proliferation and apoptosis were assessed using MTT, crystal violet staining, and Cell Death ELISA assays, respectively. Fourth, the effects of EVOO phenolic fractions on the expression levels and the activation status of HER2 oncoprotein were evaluated using HER2-specific ELISAs and immunoblotting procedures, respectively. Fifth, we preliminary addressed the molecular mechanisms underlying the anti-HER2 effects of EVOO-derived polyphenols.

We report for the first time that all the fractions containing the major EVOO polyphenols (*i.e. *the secoiridoids deacetoxy oleuropein aglycone, ligstroside aglycone, oleuropein aglycone and the lignans [+]-pinoresinol and 1-[+]-acetoxypinoresinol) can efficiently inhibit HER2 protein kinase activity by depleting the HER2 protein kinase itself. We suggest that the stereochemistry of these phytochemicals might provide an excellent and safe platform for the design of new HER2-targeted anti-breast cancer drugs.

## Methods

### Apparatus

Semi-preparative HPLC was performed with a HP 1100 series (Agilent Technologies, Palo Alto, CA, USA), equipped with a binary pump delivery system, a degasser, an autosampler, a diode array UV-VIS detector (DAD). The semi-preparative HPLC column used was a Phenomenex Luna (C_18_) column, 10 μm i.d., 25 cm × 10 mm and the flow rate was 3 mL/min.

Capillary electrophoresis (CE) were performed using a in a P/ACE™ System MDQ (Beckman Instruments, Fullerton, CA, USA). Fused-silica capillaries of 85 cm in length and 50 μm inner diameter (360 μm outer diameter) were used. CE apparatus was coupled to the mass spectrometer detector by an orthogonal electrospray interface (ESI). Mass spectrometer was a microTOF™ (Bruker Daltonik, Bremen, Germany), an orthogonal-accelerated TOF mass spectrometer (oaTOF-MS). Good sensitivity at a reasonable resolution was obtained (5,000–10,000 at 250 m/z). The trigger time was set to 50 μs, corresponding to a mass range of 50–800 m/z. Spectra were acquired by summarizing 30,000 single spectra, defining the time resolution to 1.5 s.

### Reagents, stock solutions and reference compounds

Methanol and n-hexane HPLC-grade were from Merck (Darmstadt, Germany). Distilled water with a conductivity of 18.2 MΩ was deionized by using a Milli-Q system (Millipore, Bedford, MA, USA). Oleuropein glycoside was obtained from Extrasynthèse (Genay, France). Trastuzumab (Herceptin™) – kindly provided by Hospital Universitari de Girona Dr. Josep Trueta Pharmacy (Girona, Spain) – was solubilized in bacteriostatic water for injection containing 1.1% benzyl alcohol (stock solution at 21 mg/ml), stored at 4°C and used within one month. Lapatinib (GW572016; Tykerb^®^) was gently provided by GlaxoSmithKline (GSK), Corporate Environment, Health & Safety (Brentford, Middlesex TW8 9GS UK). MG-132 was purchased from Calbiochem (Calbiochem, San Diego, CA, USA). N-acetylcysteine and Trolox were purchased from Sigma-Aldrich (St. Louis, MO, USA). Lapatinib, MG-132, and Trolox were dissolved in DMSO and stored in the dark as stock solutions (10 mM) at -20°C until utilization. NAC was dissolved in Phosphate Buffered Saline (PBS) immediately before utilization. For experimental use, trastuzumab, lapatinib, MG-132, NAC and Trolox were prepared freshly from stock solutions and diluted with growth medium. Control cells were cultured in medium containing the same concentration (*v/v*) of vehicles as the experimental cultures with treatments. The vehicle solutions had no noticeable influence on the proliferation of experimental cells.

### Sample, extraction, isolation and analysis of polyphenol fractions from EVOO

**Figure 2:**
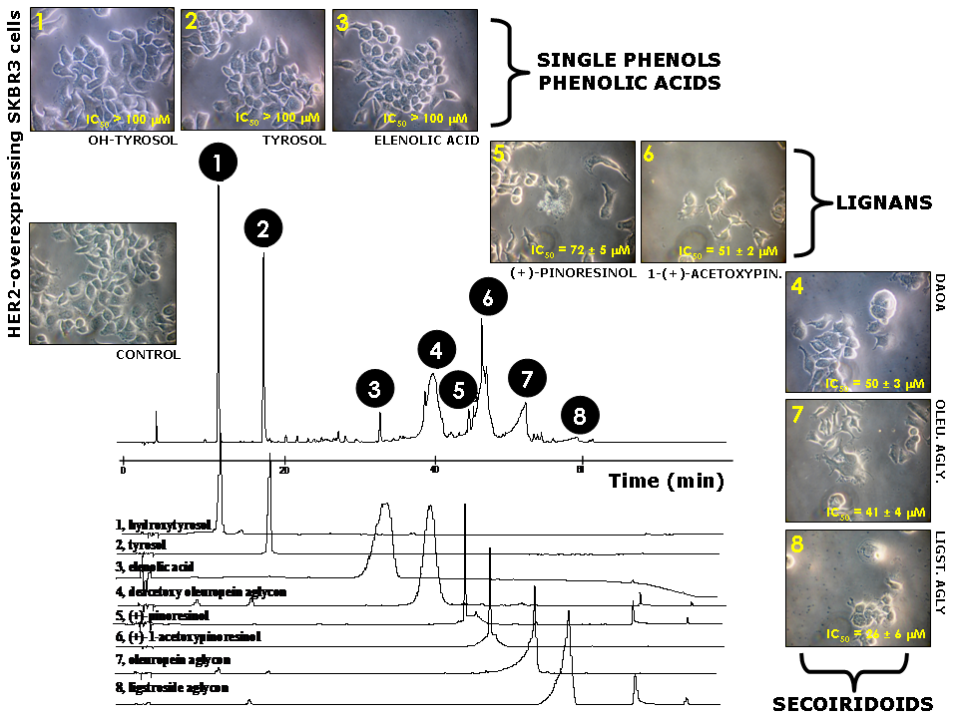
**Effects of EVOO phenolics on breast cancer cell viability**. Figure shows the chromatographic profile of an EVOO sample and the extracted ion of the main phenolics of each fraction. Detection of EVOO phenolics was performed at 280 nm (only in the analysis of elenolic acid – peak number 3 – the detection was performed at 240 nm). The metabolic status of EVOO polyphenols-treated SKBR3 cells was evaluated using a MTT-based cell viability assay and constructing dose-response curves as described in "Materials and methods". Concentrations producing the IC_50 _value (the concentration of each compound or main compound of the each fraction needed to reduce cell viability by 50% relative to untreated control cells) were calculated by interpolation. Values are means (in μM) and 95% confidence intervals (95% CI) of three independent experiments made in triplicate.

A 50/50 mixture of two commercial EVOO samples (*i.e. *Picual and Arbequina Spanish varieties) was used for this study. We have used solid phase extraction (SPE) with Diol-cartridges followed by semi-preparative reverse phase-HPLC to isolate different fractions of phenolic compounds from the EVOO. Capillary electrophoresis-electrospray ionization-mass spectrometry (CE-ESI-MS) was then employed to establish the composition of the isolated fractions because the principles of separation are completely different to semi-preparative chromatographic employed to isolate the EVOO fractions [30-37]. The identification of the phenolic compounds was obtained from the accurate mass and the isotopic pattern of the peaks applying ESI-TOF-MS analyzer. The actual composition of the isolated phenolic fractions, including experimental *m/z*, the average molecular weight and, when available, the names of the phenolic compounds, is detailed in Table 1. The fractions 1, 2, 3 and 8 mainly included a single compound (*i.e.* hydroxytyrosol, tyrosol, elenolic acid and ligstroside aglycone, respectively), while the fractions 4, 5, 6 and 7 included a mixture of several polyphenolic compounds (Figure 2). In order to simplify both the results and the discussion sections, the total composition of fractions 4, 5, 6 and 7 was expressed as the main compound contributing to each fraction (*i.e.* deacetoxyoleuropein aglycone – DAOA-, pinoresinol, acetoxypynoresinol and oleuropein aglycone, respectively). Retention times, absorption maxima, the MS data using accurate mass and analytical parameter of the HPLC-MS method are detailed in Tables 1 and 2 (Additional file 1).

**Table 1:** Composition of the isolated EVOO phenolic fractions using CE-ESI-MS

**Isolated fraction**	**Phenol composition**	
	
	**m/z experimental**	**Name**
1	153.0557	***Hydroxytyrosol***
2	137.0609	***Tyrosol***
3	241.0721	***Elenolic acid***
4	319.1171	***Deacetoxy oleuropein aglycone (DAOA)***
	393.1186	Unknown 1
	185.1176	Unknown 2
	297.1510	Unknown 3
	191.0563	Unknown 4
5	357.1135	***Pinoresinol***
	303.1225	Deacetoxy ligstroside aglycone
	361.1308	Ligstroside aglycone
6	415.1393	***Acetoxypinoresinol***
	303.1225	Deacetoxy ligstroside aglycone
	361.1285	Ligstroside aglycone
	377.1266	Oleuropein aglycone
	257.0669	Unknown 5
7	377. 1249	***Oleuropein aglycone***
	361.1286	Ligstroside aglycone
	333. 1456	Unknown 6
	287.2239	Unknown 7
	407.1363	Unknown 8
	239.0552	Unknown 9
8	361.1308	***Ligstroside aglycone***

### Cell lines and culture conditions

MCF-7 and SKBR3 breast cancer cells were obtained from the American Type Culture Collection (ATCC) and they where routinely grown in Improved MEM (IMEM; Biosource International) supplemented with 10% fetal bovine serum (FBS) and 2 mM L-Glutamine. Cells were maintained at 37°C in a humidified atmosphere of 95% air and 5% CO_2_. Cells were screened periodically for *Mycoplasma *contamination. EVOO polyphenols were prepared freshly from stock solutions and diluted with growth medium. Control cells were cultured in medium containing the same concentration (*v/v*) as the experimental cultures with treatments. The vehicle solutions had no noticeable influence on the proliferation of experimental cells.

### Construction of pBABE/HER2 retroviruses and retroviral infection of MCF-7 cells

A full-length human HER2 cDNA construct in the pCMV-SPORT6 plasmid was purchased from RZPD (Berlin, Germany). The insert was excised from pCMV-SPORT6 using *Eco*RV and *Not*I sites and blunt end ligated into the pBABE-puro retroviral vector (Addgene) at the *Eco*RI site. Retroviruses were generated by co-transfection of 293T-derived phoenix cells with the retroviral constructs (pBABE, pBABE-HER2) and the packaging vector pCL-Eco by using FuGene transfection reagent (Roche Diagnostics, Barcelona, Spain) and 5 μg of each plasmid per 0.5 × 10^6 ^cells. 293T cells were cultured at 5% CO_2_, 37°C in DMEM containing 10% (*v/v*) heat-inactivated FBS. After 48 h, medium conditioned by transfected 293T cells was filtered and immediately added to MCF-7 cells in the presence of 4 μg/ml polybrene (Sigma-Chemicals, St. Louis, MO, USA). At 48 h following infection, MCF-7/pBABE and MCF-7/HER2 cells were selected by using 2.5 μg/ml puromycin for 72 h. Expression of virally encoded HER2 protein was confirmed by HER2-specific ELISA analyses (see below).

### Metabolic status assessment (MTT-based cell viability assays)

Breast cancer cell viability was determined using a standard colorimetric MTT (3-4, 5-dimethylthiazol-2-yl-2, 5-diphenyl-tetrazolium bromide) reduction assay. Cells in exponential growth were harvested by trypsinization and seeded at a concentration of ~2.5 × 10^3 ^cells/200 μl/well into 96-well plates, and allowed an overnight period for attachment. Then the medium was removed and fresh medium along with various concentrations of EVOO phenolic fractions, trastuzumab, lapatinib, NAC, Trolox, and/or MG-132 were (concurrently or sequentially) added to cultures as specified. Control cells without agents were cultured in parallel using the same conditions with comparable media changes. Compounds were not renewed during the entire period of cell exposure. Following treatment (5 days), the medium was removed and replaced by fresh drug-free medium (100 μl/well), and MTT (5 mg/ml in PBS) was added to each well at a 1/10 volume. After incubation for 2–3 hr at 37°C, the supernatants were carefully aspirated, 100 μl of DMSO were added to each well, and the plates agitated to dissolve the crystal product. Optical Density (OD) was measured at 570 nm using a multi-well plate reader (Model Anthos Labtec 2010 1.7 reader). The cell viability effects from exposure of cells to each polyphenol fraction alone were analyzed as percentages of the control cell absorbances, which were obtained from control wells treated with appropriate concentrations of the compounds vehicles that were processed simultaneously. For each treatment, cell viability was evaluated as a percentage using the following equation:

>(OD_570 _of treated sample/OD_570 _of untreated sample) × 100.

Breast cancer cell sensitivity to agents was expressed in terms of the concentration of drug required to decrease by 50% cell viability (IC_50 _value). Since the percentage of control absorbance was considered to be the surviving fraction of cells, the IC_50 _values were defined as the concentration of agents that produced 50% reduction in control absorbance (by interpolation), respectively.

### Cell proliferation (crystal violet staining)

The ability of EVOO polyphenol compounds to affect breast cancer cells proliferation was determined using a crystal violet cell staining assay. Crystal violet is an intense stain binding to the cell nuclei and gives an OD_595 _reading that is proportional to cell number. Cells were plated and treated as described above (MTT assays). Following treatments, cells were fixed by replacing the growth medium with 100 μl/well of 4% formaldehyde in PBS (20 minutes, RT). Formaldehyde solution was removed and cells were washed twice with 200 μl/well Wash Buffer (0.1% Triton X-100 in PBS) and 2 times with 200 μl/well 1 × PBS. 100 μl of crystal violet solution was added to each well and incubated 30 minutes at RT. Cell were then washed 3 times with 200 μl/well 1 × PBS, 100 μl of 1% SDS solution was added to each well, and plates were incubated on a shaker for 1 hour at RT. Optical Density (OD) was measured at 595 nm using a multi-well plate reader (Model Anthos Labtec 2010 1.7 reader), and the cell proliferation effects from exposure of cells to each fraction were analyzed as percentages of the control cell absorbances, which were obtained from control wells treated with appropriate concentrations of the compounds vehicles that were processed simultaneously. For each treatment, cell proliferation was evaluated as a percentage using the following equation:

(OD_595 _of treated sample/OD_595 _of untreated sample) × 100.

### Apoptosis assays

The ability of EVOO-derived phenolic compounds to induce apoptosis was assessed using the Cell Death Detection ELISA^PLUS ^kit obtained from Roche Diagnostics (Barcelona, Spain). Briefly, cells (5 to 10 × 10^3^/well) were grown in 96-well plates and treated, in duplicates, for 72 h with the indicated doses of EVOO polyphenols, as specified. After treatment, the 96-well plates were centrifuged (200 × *g*) for 10 min. The supernatant was discharged, lysis buffer was added, and samples were incubated at room temperature (RT) for 30 min following the manufacturer's instructions. Anti-histone biotin and anti-DNA peroxidase antibodies were added to each well and incubated at RT for 2 h. After three washes, the peroxidase substrate was added to each well, and the plates were read at 405 nm at multiple time intervals. The enrichment of histone-DNA fragments in treated cells was expressed as fold increase in absorbance as compared with control (vehicle-treated) cells.

### Transient transfection of small interference RNAs

The small interfering RNA sequences used for targeted silencing of human HER2 were supplied by Santa Cruz Biotech (Santa Cruz, CA, USA) as double-stranded small interference RNA [Neu siRNA (h) sc-29405]. siRNA A (sc-37007), which consists of a scrambled sequence that will not lead to the specific degradation of any known cellular mRNA, was employed as negative control for experiments using HER2-targeted siRNA transfection. Transfections were performed as described in Santa Cruz technical bulletin. Briefly, cells at a confluence of 60 to 80% were transfected with the selected small interference RNAs using Santa Cruz Biotechnology's siRNA Transfection Reagent (sc-29528) and siRNA Transfection Medium (sc-36868) following the manufacturer's instructions.

### HER2-specific Enzyme-Linked Immunosorbent Assay

Determination of HER2 protein content was performed with a commercially available quantitative ELISA (Oncogene Science, Bayer Diagnostics) according to the manufacturer's protocol. To assess the effects of EVOO phenols, HER2 siRNA, NAC, and Trolox on HER2 protein concentrations, breast cancer cells, after a 24 h starvation period in media without serum, were incubated with graded concentrations of EVOO phenolics, HER2 siRNA, siRNA A, NAC or Trolox as specified. After treatment, cells were washed twice with cold-PBS and then lysed in buffer (20 mM Tris pH 7.5, 150 mM NaCl, 1 mM EDTA, 1 mM EGTA, 1% Triton X-100, 2.5 mM sodium pyrophosphate, 1 mM β-glycerolphosphate, 1 mM Na_3_VO_4_, 1 μg/ml leupeptin, 1 mM phenylmethylsulfonylfluoride) for 30 minutes on ice. The lysates were cleared by centrifugation in an Eppendorff tube (15 minutes at 14,000 × g, 4°C). Protein content was determined against a standardized control using the Pierce Protein Assay Kit (Rockford, IL, USA).

1:50, 1:500; 1: 5,000 and 1:10,000 dilutions of total cell lysates from EVOO phenols-treated, HER2 siRNA-transfected and control untreated cells were used to quantitate HER2 protein expression in cell cultures. A standard curve was generated by using standard solutions as per manufacturer's instructions. The concentrations of HER2 in test samples (in nanograms of HER2 per milligram of total protein) were determined by interpolation of the sample absorbances from the standard curve. Each experiment was performed in duplicate wells.

### Activation status of HER2

Testing for the phosphorylation (activation) status of HER2 was performed by immunoblotting procedures using the monoclonal c-*erb*B2/HER2 (phosphor-specific) antibody Ab-18 (NeoMarkers, Fremont, CA, USA). Briefly, EVOO polyphenols-treated and untreated control cells were washed twice with cold PBS and then lysed as described above. Equal amounts of protein (*i.e*. 50 μg) were resuspended in 5× Laemli sample buffer (10 min at 70°C), resolved by electrophoresis on 3–8% NuPAGE Tris-Acetate and transferred onto nitrocellulose membranes. Non-specific binding on the nitrocellulose filter paper was minimized by blocking for 1 h at RT with TBS-T buffer [25 mM Tris-HCl (pH 7.5), 150 mM NaCl, 0.05% Tween 20] containing 5% (*w/v*) nonfat dry milk. The treated filters were washed in TBS-T and then incubated with the phospho-c-*erb*B2/HER2 (clone PN2A) antibody in 5% w/v BSA, 1 × TBS-T buffer, 0.1% Tween-20 at 4°C with gentle shaking, overnight. The membranes were washed in TBS-T, horseradish peroxidase-conjugated secondary anti-mouse IgG in TBS-T was added for 1 h, and immunoreactive bands were detected by chemiluminiscence reagent (Pierce, Rockford, IL). Blots were re-probed with an antibody for β-actin to control for protein loading and transfer (data not shown). Densitometric values of proteins bands were quantified using the Scion Image software (Scion Corporation, Frederick, MD, USA).

### Statistics

Two-group comparisons were performed by the Student *t *test for paired and unpaired values. Comparisons of means of ≥ 3 groups were performed by ANOVA, and the existence of individual differences, in case of significant *F *values at ANOVA, tested by Scheffé's multiple contrasts.

## Results

### Effects of EVOO phenolic compounds on breast cancer cell viability, proliferation, and apoptosis in HER2-overexpressing SKBR3 breast cancer cells

**Figure 3:**
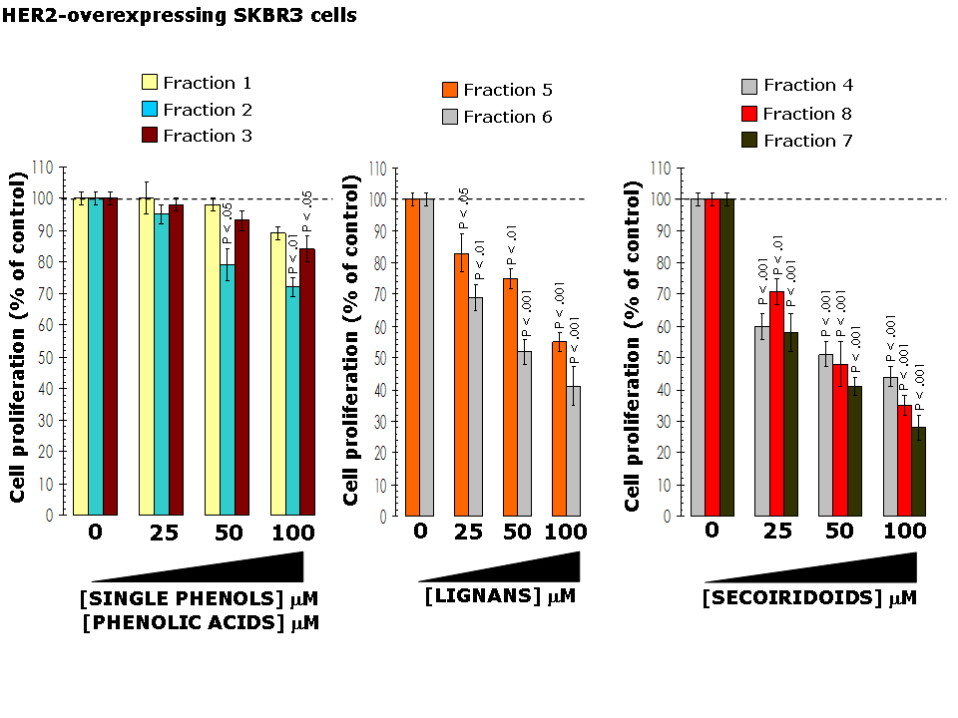
**Effects of EVOO phenolics on breast cancer cell proliferation**. SKBR3 cells were incubated with various concentrations of EVOO single phenols (*left*), fractions containing mainly EVOO lignans (*middle*) and fractions containing mainly EVOO secoiridoids (*right*) for 4 days. Cell proliferation, measured using a crystal violet assay as described in "Materials and methods", was expressed as % of untreated cells (*dashed line *= 100% cell proliferation). Results are means (columns) and 95% confidence intervals (bars) of three independent experiments made in triplicate. Statistically significant differences (one-factor ANOVA analysis) between experimental conditions and unsupplemented control cells are shown. All statistical tests were two-sided.

We initially assessed the metabolic status of HER2-overexpressing SKBR3 breast cancer cells following treatments with graded micromolar concentrations of EVOO polyphenols (6.25 → 100 μM). After 5 days of treatment, cell viability was measured using a tetrazolium salt-based (MTT) assay, and the IC_50 _value for each EVOO polyphenol was calculated as described in "Materials and methods". EVOO single phenols failed to significantly decrease SKBR3 cell viability. Thus, concentrations higher than 100 μM were needed to achieve cytotoxic responses in the presence of hydroxytyrosol, tyrosol, and elenolic acid (fractions 1, 2 and 3 respectively) (Figure 2). EVOO lignans exhibited significant cytotoxic activities against SKBR3 cells. Fraction 6, which mainly contained 1-(+)-acetoxypinoresinol was more effective than fraction 5, which mainly contained (+)-pinoresinol (IC_50s _= 51 ± 2 μM and 72 ± 5 μM, respectively) (Figure 2). Among the EVOO phenolic fractions tested, those containing the secoiridoids DAOA, oleuropein aglycone, and ligstroside aglycone were the most potent at decreasing breast cancer cell viability, with all of them exhibiting IC_50 _values lower than 50 μM (*e.g. *as low as 26 ± 6 μM for the fraction 8 mainly containing the secoiridoid ligstroside aglycone) (Figure 2). Equivalent pictures emerged when the dose-response effects of EVOO polyphenols on the proliferation rate of SKBR3 were assessed by photometry after crystal violet staining. As shown in Figure 3 (*left panel*), EVOO single phenols and polyphenol acids produced weak growth inhibitory effects in SKBR3 cells. By contrast, the fractions 5 and 6 containing EVOO lignans (Figure 3, *middle panel*) and the fractions 4, 7 and 8 containing EVOO secoiridoids (Figure 3, *right panel*) significantly inhibited SKBR3 cell proliferation in a concentration-dependent manner.

**Figure 4:**
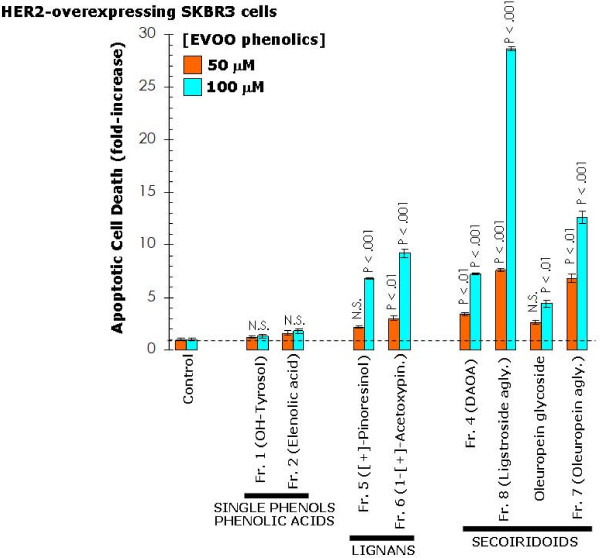
**Effects of EVOO phenolics on breast cancer apoptotic cell death**. Quantification of apoptosis-related cell death in SKBR3 cells treated with increasing concentrations of EVOO phenolics was determined by Cell Death ELISA as described in "Materials and methods". The enrichment of histone-DNA fragments EVOO polyphenols-treated cells was expressed as fold-increase in absorbance by comparing with control (vehicle-treated) cells using the following formula: [A_405 _– A_490_]_TREATED_/[A_405_-A_490_]_UNTREATED_. Data are the mean (*columns*) and 95% confidence intervals (*bars*) of three independent experiments performed in duplicate. One-factor ANOVA was used to analyze differences in the percentage of apoptosis between the various treatment groups and the control group. Statistically significant differences (one-factor ANOVA analysis) between experimental conditions and unsupplemented control cells are shown (one-factor analysis of variance). All statistical tests were two-sided. N.S.: Not statistically significant.

To evaluate whether the inhibitory effects of EVOO-derived lignans and secoiridoids on breast cancer cell viability and proliferation might actually be due to changes in apoptotic cell death, SKBR3 cells were exposed to increasing concentrations of EVOO phenolics, apoptotic cell death was measured by a Cell Death ELISA detecting apoptosis-induced DNA-histone fragmentation, and the *x*-fold increase in apoptosis was calculated by comparing the ELISA optical density readings of treated samples (with the values obtained in untreated cells as 1.0). In agreement with the results obtained in cell viability and cell proliferation assays, the lowest degree of apoptotic cell death was achieved upon treatment with hydroxytyrosol and elenolic acid (Figure 4). Treatment with the lignans-rich fractions 5 and 6 notably increased (up to ~10-fold at 100 μM 1-(+)-acetoxypinoresinol) the apoptotic cell death of SKBR3 cells (Figure 4). SKBR3 cells were likewise exquisitely sensitive to secoiridoids-rich fractions 4, 7 and 8 as they exhibited the highest degree of apoptotic cell death following exposure to fraction 4 (DAOA, > 5-fold increase), fraction 8 containing (ligstroside aglycone, > 25-fold increase), and fraction 7 (oleuropein aglycone, > 10-fold increase) (Figure 4). Interestingly, the precursor form oleuropein glycoside was significantly less effective than its oleuropein aglycone derivative, thus revealing that the glucose moiety in oleuropein structure strongly affects its anti-breast cancer activity (Figure 4).

### EVOO phenolics preferentially inhibit the proliferation of HER2-overexpressing breast cancer cells

**Figure 5:**
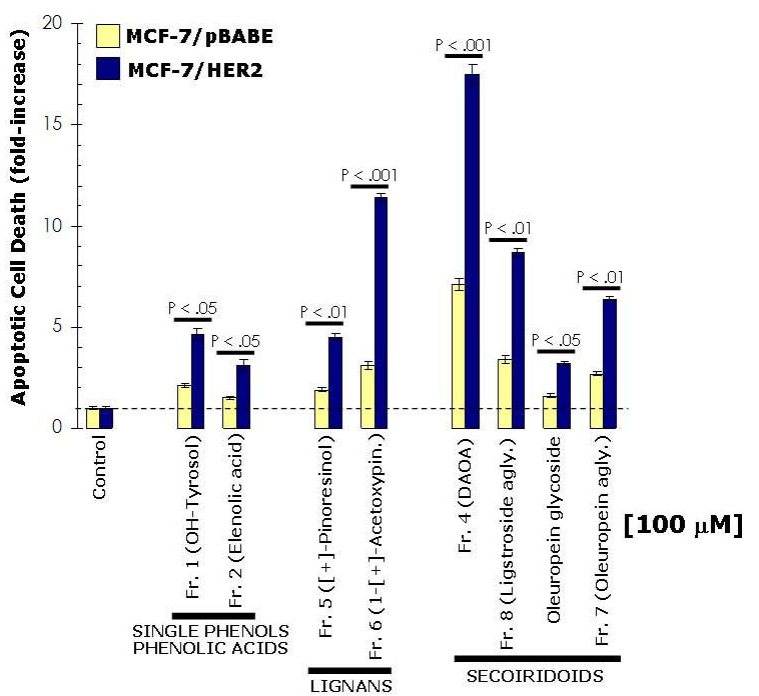
**Apoptotic effects of EVOO phenolics in MCF-7 breast cancer cells engineered to overexpress HER2**. Quantification of apoptosis-related cell death in MCF-7/HER2 and MCF-7/pBABE matched control cells treated with increasing concentrations of EVOO phenolics was determined by Cell Death ELISA as described in "Materials and methods". The enrichment of histone-DNA fragments in EVOO polyphenols-treated cells was expressed as fold-increase in absorbance by comparing with control (vehicle-treated) cells using the following formula: [A_405 _– A_490_]_TREATED_/[A_405_-A_490_]_UNTREATED_. Data are the mean (*columns*) and 95% confidence intervals (*bars*) of three independent experiments performed in duplicate. One-factor ANOVA was used to analyze differences in the percentage of apoptosis between HER2-negative MCF-7/pBABE cells and HER2-positive MCF-7/HER2 cells. Statistically significant differences are shown (one-factor ANOVA analysis). All statistical tests were two-sided.

**Figure 6:**
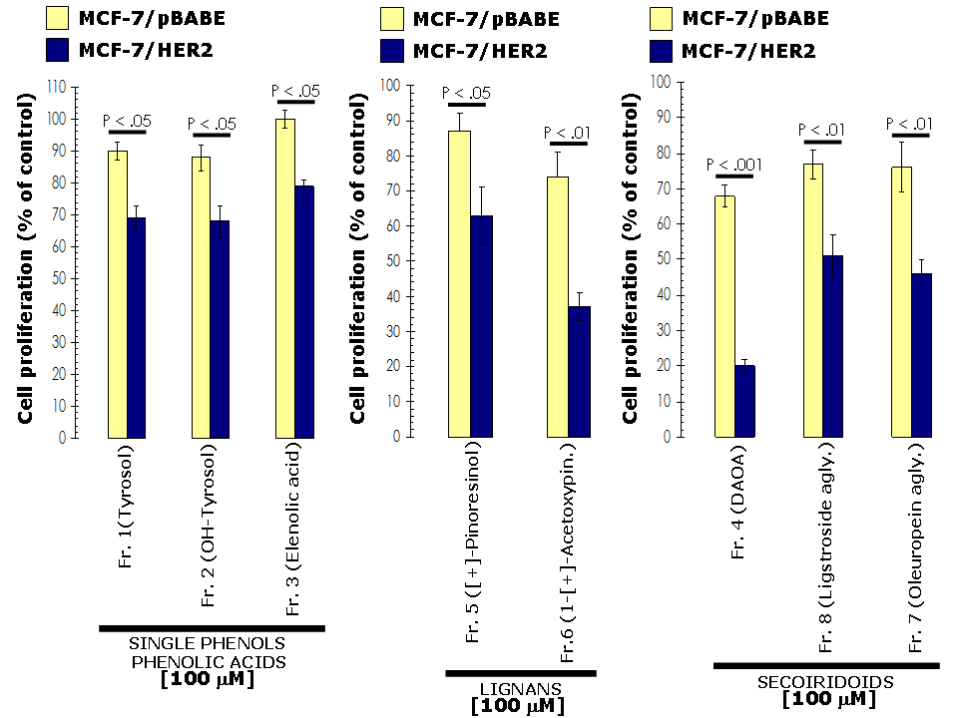
**Effects of EVOO phenolics on HER2-enhanced breast cancer cell proliferation**. MCF-7/HER2 cells and MCF-7/pBABE matched control cells were incubated with 100 μM of EVOO single phenols (*left*), fractions containing mainly EVOO lignans (*middle*) and fractions containing mainly EVOO secoiridoids (*right*) for 4 days. Cell proliferation, measured using a crystal violet assay as described in "Materials and methods", was expressed as % of untreated cells (*dashed line *= 100% cell proliferation). Results are means (*columns*) and 95% confidence intervals (*bars*) of three independent experiments made in triplicate. Statistically significant differences (one-factor ANOVA analysis) between HER2-negative MCF-7/pBABE control cells and HER2-positive MCF-7/HER2 cells are shown. All statistical tests were two-sided.

SKBR3 cells represent a widely used breast cancer *in vitro *model characterized by exhibiting natural *HER2 *gene amplification, HER2 receptor protein overexpression and HER2-dependency for cell proliferation and survival [38,39]. To evaluate whether EVOO polyphenols preferentially exhibit tumoricidal effects against HER2-overexpressing breast cancer cells, we further explored both the apoptotic/anti-proliferative effects of EVOO polyphenols in MCF-7 breast cancer cells – which express physiological levels of HER2 (*i.e. *one single copy of HER2 gene) and in MCF-7 cells stably transduced with pBABE-HER2 or pBABE (empty control) retroviral vectors. The level of HER2 overexpression achieved in MCF-7/(pBABE)HER2 cells (~70-fold increase when compared to MCF-7/pBABE matched control and MCF-7 parental cells) was comparable to that reported in MCF-7/Her2-18 cells, a well-characterized MCF-7-derived HER2-overexpressing clone engineered to stably express the full-length human HER2 cDNA controlled by a SV40 viral promoter [40]. Forced expression of HER2 in MCF-7 cells increased by > 4-fold the apoptotic effects of the fraction 6 containing the EVOO lignan 1-(+)-acetoxypinoresinol. High levels of HER2 promoted also exacerbated apoptotic responses to fractions 4, 7 and 8 containing mainly EVOO secoiridoids DAOA, ligstroside aglycone, and oleuropein aglycone, respectively. For example, treatment with the DAOA-rich fraction 4 increased apoptotic cell death by 17.5-times and 7.1-times in HER2-overexpressing MCF-7/HER2 transfectants and in HER2-negative MCF-7/pBABE matched control cells, respectively (Figure 5). The notion that HER2 overexpression in breast cancer cells significantly decreases the capability of human breast epithelial cells to overcome EVOO polyphenols-induced cell injuries was further supported when analyzing the anti-proliferative effects of EVOO polyphenols in MCF-7/pBABE and MCF-7/HER2 cells. As shown in Figure 6 (*left panel*), EVOO single phenols and EVOO polyphenol acids were ineffective against HER2-negative MCF-7/pBABE cells while they exhibited weak growth-inhibitory effects against HER2-overexpressing MCF-7/HER2 cells. Remarkably, the fraction 6 containing mainly the EVOO lignan 1-(+)-acetoxypinoresinol reduced the proliferation rate of MCF-7/HER2 cells by 63%, whereas this reduction was as low as 26% in MCF-7/pBABE matched control cells (Figure 6, *middle panel*). All the EVOO secoiridoids exhibited slight anti-proliferative effects in HER2-negative MCF-7/pBABE cells. Conversely, HER2-overexpressing MCF-7/HER2 cells were exquisitely sensitive to the growth-inhibitory effects of DAOA-rich fraction 4 (~80% inhibition of cell proliferation at 100 μM), ligstroside aglycone-rich fraction 8 (~50% inhibition of cell proliferation at 100 μM) and oleuropein aglycone-rich fraction 7 (~55% inhibition of cell proliferation at 100 μM) (Figure 6, *right panel*). Overall, these results suggest that EVOO polyphenols preferentially suppress the growth of HER2-overexpressing breast cancer cells.

### EVOO polyphenols deplete HER2 oncoprotein

**Figure 7:**
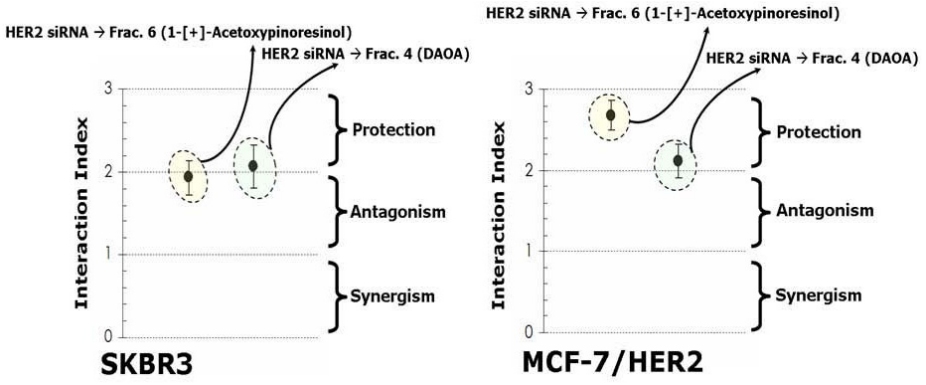
**Effects of HER2 siRNA on EVOO polyphenols-induced cytotoxicity**. ~5 × 10^3 ^cells (96-well plates) overnight serum-starved SKBR3 (*left*) and MCF-7/HER2 (*right*) cells were transfected with HER2 siRNA or control siRNA (80 pmols/well) before exposure to 25 μM 1-[+]-acetoxypinoresinol or 25 μM DAOA. The metabolic status of SKBR3 and MCF-7/HER2 cells was evaluated using a MTT-based cell viability assay and Interaction Indexes were calculated by dividing expected cytotoxicities (additive models: sum of cell toxicities induced by each agent alone) by those obtained experimentally in the actual combination (Interaction Indexes < 1, = 1, > 1 < 2, and > 2 denote a nature of the interaction synergistic, additive, antagonistic and protective, respectively). Results are means (*dots*) and 95% confidence intervals (*bars*) of three independent experiments made in triplicate.

To evaluate whether, in terms of cell viability, the biological activity of EVOO polyphenols did require HER2 overexpression as a necessary molecular "hallmark", we used a siRNA HER2 depletion approach to determine the effect of "HER2 protein-dose" on the sensitivity of breast cancer cells to EVOO polyphenols. SKBR3 and MCF-7/HER2 cells were transfected with 80 pmols of HER2 siRNA or control siRNA (*i.e. *siRNA A). 72 h after HER2-targeted siRNA transfection, HER2 protein levels decreased up to ~65-70% of those present in SKBR3 and MCF-7/HER2 cells transfected with a control siRNA (Figure 1, Additional file 1). Interestingly, a combination of sequential HER2 siRNA followed by treatment with sub-optimal doses of the 1-[+]-acetoxypinoresinol-rich fraction 6 and of the DAOA-rich fraction 4 strikingly demonstrated an antagonistic to protective nature of the cytotoxic interaction (Interaction Indexes > 2.0; Figure 7).

**Figure 8:**
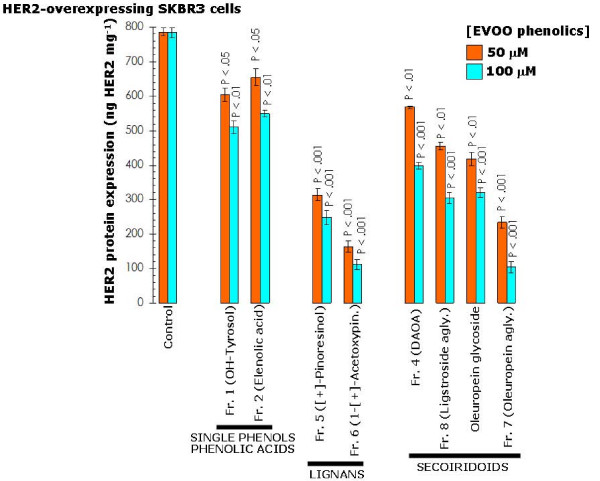
**Influence of EVOO phenolics on the levels of HER2 oncoprotein in SKBR3 breast cancer cells**. Overnight serum-starved SKBR3 cells were cultured in DMEM medium-0.1% FBS in the absence or presence of increasing concentrations of EVOO phenolics for 48 h. The Oncogene Science HER2 microtiter ELISA was used according to the manufacturer's instructions to compare HER2 protein concentrations in whole cell lysates from EVOO polyphenols-treated and untreated control cells. Results are means (*columns*) and 95% confidence intervals (*bars*) of three independent experiments made in triplicate. Statistically significant differences between experimental conditions and unsupplemented control cells are shown (one-factor ANOVA analysis). All statistical tests were two-sided.

**Figure 9:**
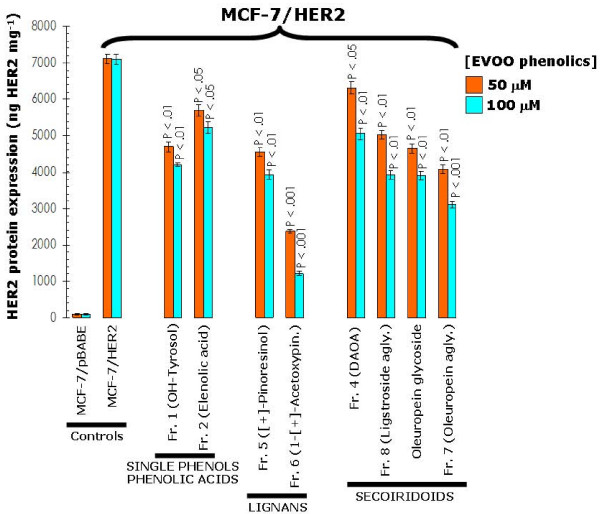
**Influence of EVOO phenolics on the levels of HER2 oncoprotein in MCF-7/HER2 breast cancer cells**. Overnight serum-starved MCF-7/HER2 cells were cultured in DMEM medium-0.1% FBS in the absence or presence of increasing concentrations of EVOO phenolics for 48 h. The Oncogene Science HER2 microtiter ELISA was used according to the manufacturer's instructions to compare HER2 protein concentrations in whole cell lysates from EVOO polyphenols-treated and untreated control cells. Results are means (*columns*) and 95% confidence intervals (*bars*) of three independent experiments made in triplicate. Statistically significant differences between experimental conditions and unsupplemented control cells are shown (one-factor ANOVA analysis). All statistical tests were two-sided.

The fact that depletion of endogenous HER2 significantly prevented the growth-inhibitory effects of EVOO polyphenols together with our earlier findings demonstrating that the down-regulatory effects of the secoiridoid oleuropein aglycone were specifically restricted to HER2 without affecting other key members of the oncogenic HER network such as HER1 (EGFR) [20], suggested that secoiridoids- and lignans-induced changes in cell viability might relate, at least in part, to changes in the expression of HER2 oncoprotein. To evaluate this hypothesis we examined the effects of long-term treatments (*i.e. *48 hours) with graded concentrations of EVOO polyphenols on the naturally-occurring overexpression of HER2 protein in SKBR3 cells (Figure 8). At its highest concentration (*i.e. *100 μM), the EVOO single phenol hydroxytyrosol slightly reduced HER2 protein expression by 35%. Remarkably, HER2 expression was drastically reduced by 68% and 86% in the presence of 100 μM of the fractions 5 and 6 containing mainly EVOO lignans (+)-pinoresinol and 1-(+)-acetoxypinoresinol, respectively. Similarly to EVOO lignans, EVOO secoiridoids were very effective at inhibiting HER2 expression, with inhibitory percentages ranging from 50%–60% in the presence of DAOA-rich fraction 4 and ligstroside aglycone-rich fraction 8 to 87% inhibition upon treatment with the oleuropein aglycone-rich fraction 7. We then evaluated if the growth inhibitory effects of EVOO polyphenols against MCF-7/HER2 cells were also accompanied with changes in the expression levels of HER2 oncoprotein (Figure 9). The EVOO single phenol hydroxytyrosol notably decreased HER2 protein expression by 41%, while the fraction 6 containing mainly the EVOO lignan 1-(+)-acetoxypinoresinol drastically reduced HER2 expression by 83%. All the EVOO secoiridoids significantly reduced HER2 expression, from ~30% inhibition in the presence of DAOA-rich fraction 4 to 56% inhibition in the presence of the oleuropein aglycone-rich fraction 7. These findings reveal for the first time that all the major complex phenols present in EVOO (*i.e. *secoiridoids and lignans) drastically suppress HER2 oncoprotein overexpression in human breast cancer cells. Neither secoiridoids nor lignans treatments caused detectable changes in HER1 (EGFR) expression in breast cancer cells (data not shown).

### EVOO polyphenols inhibit HER2 tyrosine phosphorylation

**Figure 10:**
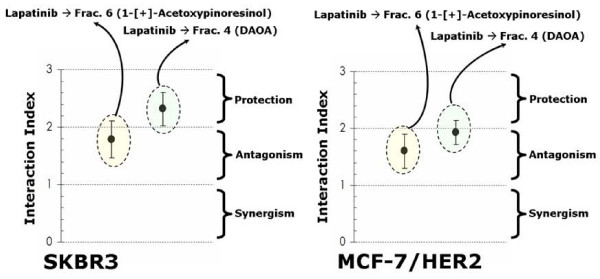
**Influence of the activation (phosphorylation) status of HER2 tyrosine kinase activity on the cytotoxic activity of EVOO polyphenols**. ~5 × 10^3 ^cells (96-well plates) overnight serum-starved SKBR3 (*left*) and MCF-7/HER2 (*right*) cells were treated with 0.1 μM lapatinib before exposure to 25 μM 1-[+]-acetoxypinoresinol or 25 μM DAOA. The metabolic status of treated and untreated control cells was evaluated using a MTT-based cell viability assay and Interaction Indexes were calculated by dividing expected cytotoxicities (additive models: sum of cell toxicities induced by each agent alone) by those obtained experimentally in the actual combination (Interaction Indexes < 1, = 1, > 1 < 2, and > 2 denote a nature of the interaction synergistic, additive, antagonistic and protective, respectively). Results are means (*dots*) and 95% confidence intervals (*bars*) of three independent experiments made in triplicate.

**Figure 11:**
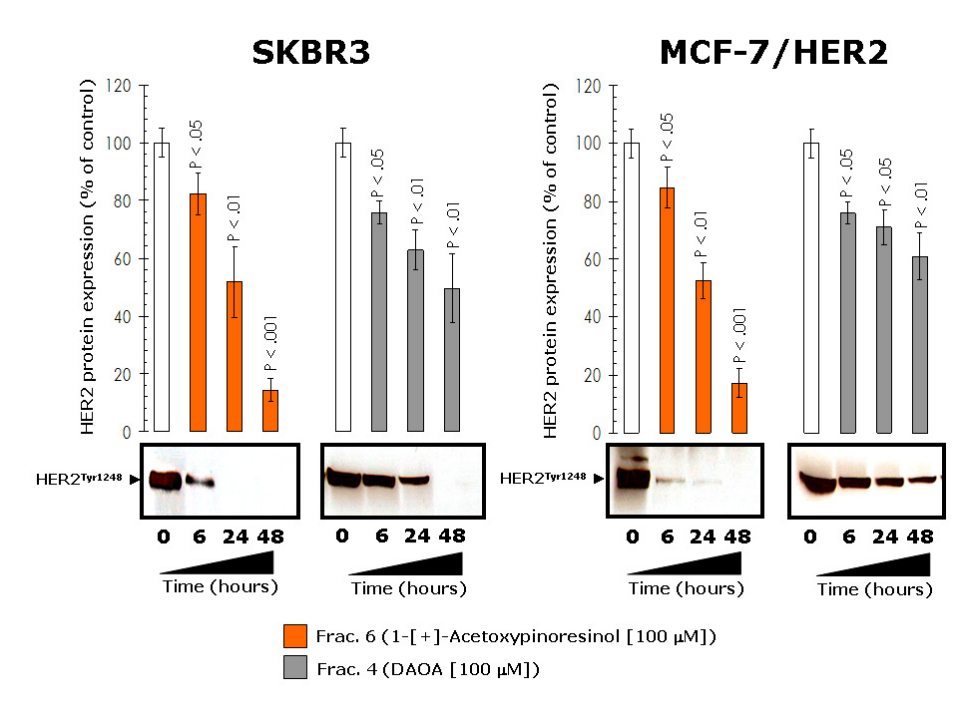
**Influence of EVOO polyphenols on the levels of HER2 tyrosine kinase activity**. Overnight serum-starved SKBR3 (*left*) and MCF-7/HER2 (*right*) cells were treated with 100 μM 1-[+]-acetoxypinoresinol and 100 μM DAOA for 0, 6, 24 and 48 hr. Oncogene Science HER2 microtiter ELISA was used according to the manufacturer's instructions to compare HER2 protein concentrations in whole cell lysates throughout the time-course. HER2 expression at the 0 hr-time point was set as 100%. Results are means (*columns*) and 95% confidence intervals (*bars*) of three independent experiments made in triplicate. Statistically significant differences between experimental conditions and unsupplemented control cells at the 0 hr-time point are shown (one-factor ANOVA analysis). All statistical tests were two-sided. EVOO polyphenols-treated and untreated control cells (0 hr) were tested in parallel for p185^HER2 ^autophosphorylation at Tyr1248 using immunoblotting procedures as described in "Materials and methods".

Since apigenin and luteolin, two naturally occurring flavonoids structurally related to EVOO polyphenols, have been found to inhibit tyrosine phosphorylation and deplete HER2 protein through HER2 protein degradation [41-43], we envisioned that an analogous posttranslational mechanism may also contribute to the anti-HER2 actions of EVOO polyphenols. First, to evaluate whether the biological activity of EVOO polyphenols in terms of cell viability not only related to HER2 protein overexpression but further required an enhanced tyrosine kinase activity of HER2 as a molecular "hallmark" in EVOO polyphenols-sensitive breast cancer cells, we used a pharmacological approach to determine the effect of "HER2 activity-dose" on the tumoricidal effects of EVOO polyphenols. Similarly to siRNA HER2-induced depletion of HER2 protein, specific blockade of HER2 constitutive hyperactivation (autophosphorylation at Tyr1248; Figure 2, Additional file 1) using the dual-HER1/HER2 tyrosine kinase inhibitor lapatinib (Tykerb™) was sufficient to preclude any further cytotoxic action of EVOO polyphenols. Thus, a combination of sequential lapatinib (0.1 μM) followed by treatment with sub-optimal doses of the 1-[+]-acetoxypinoresinol-rich fraction 6 and DAOA-rich fraction 4 demonstrated an antagonistic to protective nature in their cytotoxic interactions (Interaction Indexes > 2.0; Figure 10). To investigate the kinetics of inhibition/depletion of HER2 activity/expression, we treated SKBR3 and MCF-7/HER2 cells with EVOO polyphenols for different time periods and harvested them for ELISA-based analysis of HER2 protein expression. HER2 tyrosine autophosphorylation levels (*i.e. *activation status of HER2 Tyr1248) were measured by immunoblotting procedures. The HER2 protein levels decreased in a time-dependent manner following exposure to EVOO polyphenols, with significant HER2 depleting effects occurring as early as 6 hr after treatment with the fraction 6 containing mainly 1-[+]-acetoxypinoresinol (Figure 11). Fraction 6-decreased HER2 protein led to a coordinate decrease in HER2 autophosphorylation, which significantly declined after 6 hr and was barely detectable after 24 hr (Figure 11). Although less markedly than fraction 6, the fraction 4 containing mainly the EVOO secoiridoid DAOA also decreased HER2 protein expression and HER2 tyrosine kinase activity in a time-dependent manner, with the most significant anti-HER2 expression/activity effects occurring after 48 hr (Figure 11). These findings, altogether, strongly suggest that EVOO polyphenols could inhibit HER2 protein kinase activity by depleting the HER2 protein kinase itself.

### EVOO polyphenols-induced depletion of HER2 protein depends on proteasomal degradation but does not relate to anti-oxidant effects

The finding that all the major complex phenols present in EVOO (*i.e. *lignans and secoiridoids) exhibit the ability to drastically down-regulate HER2 protein regardless of the molecular mechanism contributing to HER2 overexpression (*i.e*. naturally by gene amplification in SKBR3 cells and ectopically driven by a viral promoter in MCF-7 cells transduced with the human HER2 cDNA) suggested that the anti-HER2 effects of EVOO polyphenols did not relate to factors controlling the rate of HER2 gene promoter-regulated HER2 *de novo *synthesis [44,45]. Accordingly, HER2 mRNA levels did not significantly decline even after 24 hours after treatment with EVOO polyphenols (data not shown). To better delineate the mechanism of EVOO polyphenols-mediated HER2 down-regulation, we tested the following hypotheses: 1.) Since all the main polyphenols that occur at high levels in EVOO have demonstrated antioxidant activity and antioxidants are believed to be responsible for a number of EVOO's biological activities, we envisioned that EVOO polyphenols-induced depletion of HER2 might represent a general response of HER2-overexpressing cancer cells growing upon anti-oxidant conditions. To test this hypothesis MCF-7/HER2 cells and MCF-7/pBABE matched control cells were treated with graded concentrations of the well-established anti-oxidants 6-hydroxy-N-acetylcysteine (NAC, a glutathione precursor and scavenger of reactive oxygen species) and 2,5,7,8-tetramethylchroman-2-carboxylic acid (Trolox, a water-soluble vitamin E analogue). Interestingly, MCF-7/HER2 cells were more sensitive to the anti-proliferative effects of NAC (Figure 3; Additional file 1) and Trolox (Figure 4; Additional file 1) as they exhibited IC_50 _values significantly lower (3 to 4-times) than those found in MCF-7/pBABE cells. However, treatment with either Trolox or NAC failed to modulate HER2 protein levels in MCF-7/HER2 cells (Figures 3 and 4; Additional file 1). Overall, these results suggest that anti-oxidant agents preferentially suppress the growth of HER2-overexpressing cancer cells, whereas promoting an antioxidant status in HER2-positive breast cancer cells is not sufficient to down-regulate HER2 expression in human breast cancer cells.

**Figure 12:**
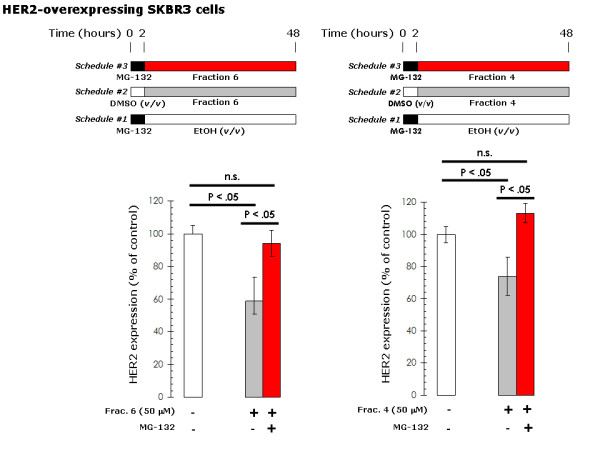
**Effects of proteasome inhibition on EVOO polyphenols-induced down-regulation of HER2 protein in breast cancer cells**. MCF-7/HER2 cells were exposed to three different schedules: 1.) 1 μM MG-132 (2 hr) → EVOO polyphenols (up to 48 hr); 2.) DMSO (*v/v*; 2 hr) → EVOO polyphenols (up to 48 hr); 3.) 1 μM MG-132 (2 hr) → EtOH (*v/v*; up to 48 hr). Oncogene Science HER2 microtiter ELISA was used according to the manufacturer's instructions to compare HER2 protein concentrations in whole cell lysates from schedules #1, #2, and #3. HER2 expression in untreated control cells was set as 100%. Results are means (*columns*) and 95% confidence intervals (*bars*) of three independent experiments made in triplicate. Statistically significant differences between experimental conditions and unsupplemented control cells are shown (one-factor ANOVA analysis).

**Figure 13:**
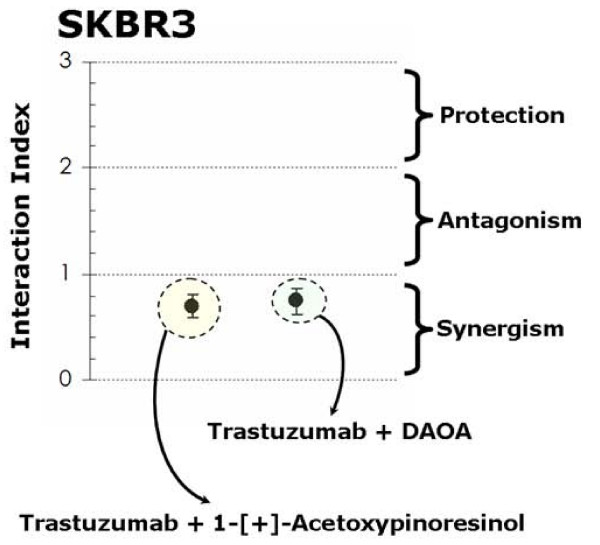
**Synergy analysis of the concurrent combination with EVOO polyphenols and the anti-HER2 monoclonal antibody trastuzumab in SKBR3 breast cancer cells**. ~5 × 10^3 ^cells (96-well plates) overnight serum-starved SKBR3 cells were treated with 100 μg/ml trastuzumab in the absence or presence of 25 μM 1-[+]-acetoxypinoresinol or 25 μM DAOA. The metabolic status of treated and untreated control cells was evaluated using a MTT-based cell viability assay and Interaction Indexes were calculated by dividing expected cytotoxicities (additive models: sum of cell toxicities induced by each agent alone) by those obtained experimentally in the actual combination (Interaction Indexes < 1, = 1, > 1 < 2, and > 2 denote a nature of the interaction synergistic, additive, antagonistic and protective, respectively). Results are means (*dots*) and 95% confidence intervals (*bars*) of three independent experiments made in triplicate.

Since the ability of EVOO polyphenols to deplete HER2 protein did not appear to relate to their impact on the antioxidant status of breast cancer cells, we then speculated that EVOO secoiridoids and lignans might deplete HER2 through proteolysis, the molecular mechanism through which other structurally related plant polyphenols such as the flavonoids apigenin and luteolin suppress HER2 protein expression in breast cancer cells [41-43]. A role of proteasomal degradation in EVOO polyphenols-mediated HER2 down-regulation was likewise supported by the fact that pre-treatment (2 hr) with the proteasome inhibitor MG-132 fully prevented the reduction of HER2 protein levels induced by EVOO polyphenols (Figure 12). To further corroborate the suggestion that EVOO polyphenols induce cell growth inhibition by depleting HER2 protein in HER2-overexpressing breast cancer cells *via *proteasomal degradation, we analyzed whether supra-additive or synergistic interactions occurred upon concurrent treatment with EVOO polyphenols and anti-HER2 agents that promote HER2 down-regulation through proteasome-independent mechanisms [12,46,47]. Simultaneous exposure of SKBR3 cells to the anti-HER2 monoclonal antibody trastuzumab with sub-optimal doses of the 1-(+)-acetoxypinoresinol-rich fraction 6 or the DAOA-rich fraction 4 likewise demonstrated greater decreases in cell viability that did each agent alone (Interaction Indexes < 1.0; Figure 13). This synergistic interaction occurring between EVOO-derived lignans and secoiridoids further extends our previous findings that demonstrated a supra-additive cytotoxic effect when combining trastuzumab with the EVOO secoiridoid oleuropein aglycone [20].

## Discussion

The ultimate molecular mechanism(s) determining how specific components of EVOO may influence the genetic program that drives breast cancer development and progression remain(s) largely obscure. Our experimental approach in the last few years has suggested that EVOO-related anti-breast cancer actions mainly affect the occurrence, the aggressive behavior and the therapeutic management of *HER2 *oncogene-driven breast carcinomas. First, when we used one of the most useful *in vivo *carcinogenesis systems of breast cancer – that of the DMBA-induced mammary carcinogenesis in female Sprague-Dawley rats, in which stepwise molecular analysis of the transformation process is conducted from the very early to the terminal stages of tumor development – a high EVOO diet was found to act as a negative modulator of the mammary carcinogenesis induced by genotoxic agents, conferring to the tumors mainly an indolent clinical behavior and a histopathological pattern compatible with a lower degree of malignancy [13,14]. Mechanistically, we demonstrated that high EVOO diet influenced negatively experimental mammary carcinogenesis modifying the mRNA expression levels of *neu *(*HER2*) [14]. Second, when using human breast cancer-derived *in vitro *models naturally exhibiting *HER2 *gene amplification and HER2 protein overexpression, exogenous supplementation with physiological concentrations of oleic acid (OA; 18:1n-9) – the main ω-9 monounsaturated fatty acid (MUFA) in EVOO – was found to drastically suppress the expression of HER2 and to synergistically enhance both the growth-inhibitory and the HER2 down-regulatory effects of the monoclonal antibody trastuzumab (Herceptin™) [15-17]. The above findings generated intense public interest, since no toxicities have been reported or suspected with OA, and suggested that supplementation with EVOO might represent a promising dietary intervention aimed to prevent and/or manage HER2-related carcinomas. However, it should be noted that EVOO consists primarily of triacylglycerols rich in the ω-9 MUFA OA and non-glyceridic constitutes comprising approximately 0.5% to 1% EVOO, which include at least 30 phenolic compounds. Although we recently presented evidence that the aglycone form of oleuropein – a member of the secoiridoid family of EVOO polyphenols that has mainly been implicated in the organoleptic characteristics of EVOO such as bitterness – significantly enhances the efficacy of anti-HER2 therapeutics in cultured HER2-overexpressing breast cancer cells when compared to simple phenols (*e.g. *tyrosol, hydroxytyrosol) [20], the anti-breast cancer effects of other EVOO-derived polyphenols such as the lignan (+)-1-acetoxypinoresinol or the secoiridoids deacetoxy oleuropein aglycone and ligstroside aglycone remained to be addressed.

We now provide new insights on the mechanisms by which good-quality OO, *i.e. *polyphenols rich-EVOO, may contribute to lower breast cancer risk in a HER2-dependent manner. First, EVOO phenolic compounds with a simple structure, involving only a single phenol ring might be incapable to exert strong anti-breast cancer actions. Indeed, a more complex (*i.e. *polyphenolic) structure appears to be required in order to exert these effects. Thus, both the EVOO-derived single phenols tyrosol and hydroxytyrosol and the EVOO-derived polyphenol acid elenolic acid exhibited significantly lower anti-proliferative and pro-apoptotic effects than those observed with the EVOO fractions rich in the complex polyphenols lignans and secoiridoids. Second, when we investigated the tumoricidal effects of EVOO polyphenols against breast epithelial cells bearing different endogenous levels of the *HER2 *oncogene, it became clear that high levels of HER2 oncoprotein constitute a molecular feature through which EVOO polyphenols exert, at least in part, their anti-breast cancer actions. EVOO polyphenols differentially induced breast cancer cell growth inhibition by promoting apoptotic cell death in HER2-positive breast cancer cells, with marginal tumoricidal effects occurring in HER2-negative breast cancer cells. Moreover, we found that EVOO polyphenols repressed the phosphor-Tyrosine levels of HER2 and also depleted HER2 protein levels. The definite mode of action underlying EVOO polyphenols-induced blockade of HER2 tyrosine kinase activity and down-regulation of HER2 protein expression was beyond the scope of this study. Nevertheless, when considering that the primary mechanism driving HER2 levels in human breast cancer cells is gene amplification and overexpression under the control of the endogenous promoter [44,45], the fact that EVOO polyphenols drastically decreased HER2 protein content regardless the molecular mechanism contributing to HER2 overexpression strongly suggests that EVOO polyphenols do not significantly affect the cellular transcriptional machinery that controls the endogenous *HER2 *locus in breast cancer cells. Mechanistically, and unlike the EVOO ω-9 MUFA OA – which has been found to *indirectly *suppress the transcriptional activity of *HER2 *gene by up-regulating the transcriptional repressor PEA3 and inducing formation of inhibitory "PEA3 transcription factor-PEA3 DNA binding site" complexes at the HER2 promoter – [18,19] the anti-HER2 effects of EVOO polyphenols appear to relate to the inhibition of HER2 tyrosine kinase activity as a result of HER2 protein depletion. This notion is supported by the following findings: 1.) EVOO polyphenols-induced inhibition of HER2 expression does not reflect a wider and unspecific inhibitory effect against other HER members closely related to HER2 in terms of structure and activity [20]; 2.) siRNA-induced depletion of HER2 protein protects breast cancer cells against EVOO-induced cell growth inhibition, thus suggesting that EVOO polyphenols should interact necessarily with the HER2 protein itself to trigger their mechanism of action; 3.) blocking ATP from binding to the tyrosine kinase (TK) domain of HER2 upon treatment with a potent, reversible, selective dual-HER1/HER2 TK inhibitor lapatinib prevents EVOO polyphenols-induced inhibition of breast cancer cell growth, thus suggesting that EVOO lignans and secoiridoids may function as phosphotyrosine-receptor kinase blockers by competing with ATP; and 4.) pre-treatment of HER2-overexpressing breast cancer cells with the proteasome inhibitor MG-132 blocks the depletion of HER2 protein induced by EVOO polyphenols, thus suggesting that EVOO-derived lignans and secoiridoids can efficiently inhibit HER2 protein kinase activity by depleting the HER2 protein kinase itself. Therefore, it is reasonable to suggest that EVOO polyphenols directly affect HER2 levels by promoting proteasomal degradation of HER2 as recently described for other structurally-related naturally-occurring polyphenols such as the flavonoids apigenin and luteolin [41-43].

Although our current findings provide new insights on the mechanisms by which good-quality OO, *i.e. *polyphenols rich-EVOO, might contribute to lower breast cancer risk in a HER2-dependent manner, extreme caution must be applied when extrapolating *in vitro *results into clinical practice. One obvious limitation of our current results is that the phenolics that were active (*i.e. *lignans and secoiridoids) exhibited tumoricidal effects against cultured breast cancer cells at concentrations (> 50 μM) that are unlikely to be achieved *in vivo *[46-48]. Indeed, it has to be clarified if these compounds will be accessible in the breast tumor tissue *in vivo*. In this regard, an important step in the body metabolism might be that EVOO polyphenols rapidly split into inactive compounds. In this regard, the secoiridoid oleuropein aglycone is split into hydroxytyrosol or tyrosol and elenolic acid [46-50], both of them notably less effective than the parental secoiridoid in terms of cytotoxicity and HER2 down-regulation. Nevertheless, EVOO-derived lignans may represent a different molecular scenario when compared to EVOO-derived secoiridoids. Saarinen *et al. *recently evaluated the accessibility and accumulation of lignans to breast cancer tissue after their oral administration to athymic mice bearing MCF-7 human breast cancer tumors [51]. Importantly, tumor tissue accumulated up to 92% of the lignans levels found in serum, thus suggesting that the anticancer activity of lignans may be due to their direct local effects on the breast cancer tissues. Of note, a randomized double-blind placebo-controlled clinical trial recently evaluated the effects of dietary flaxseed, which has an exceptionally high concentration of lignans on tumor biological markers in postmenopausal patients with newly diagnosed breast cancer [52]. The results of this clinical trial demonstrated that daily intake of 25 g flaxseed can significantly reduce cell proliferation, increase apoptosis, and affect cell signaling by reducing HER2 expression of breast tumors. A 71.0% reduction in HER2 expression and an increase in apoptosis (30.7%) were observed in the flaxseed, but not in the placebo group. In fact, the total intake of flaxseed correlated with changes in HER2 expression and apoptotic index [52].

## Conclusion

**Figure 14:**
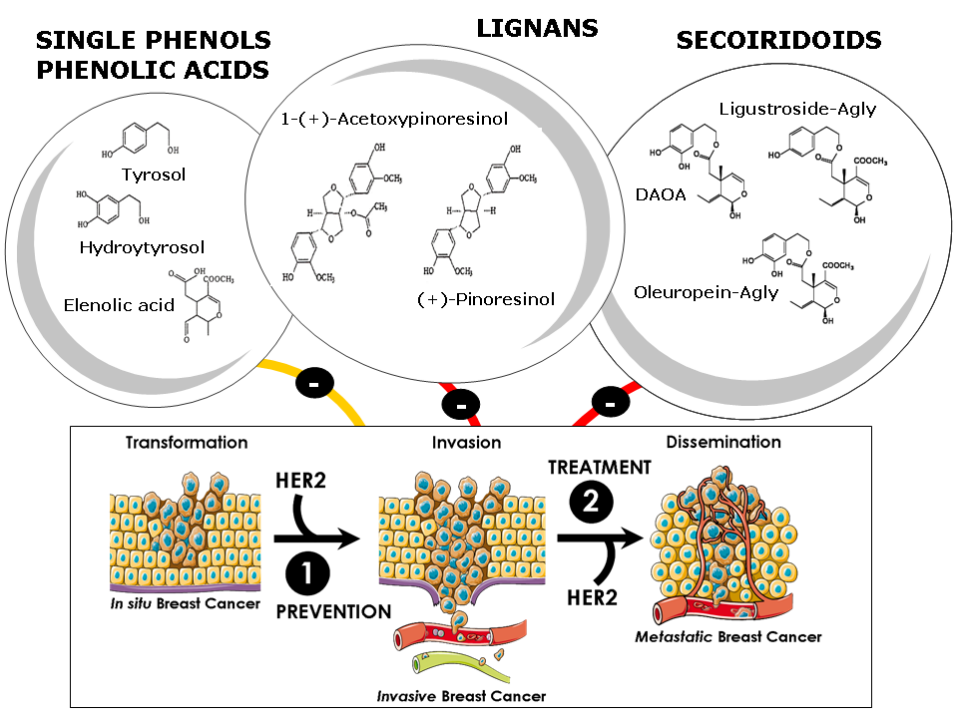
**EVOO phenolics, HER2, and breast cancer progression: Therapeutic opportunities**. Although an appropriate dietary intervention reproducing the prominent anti-HER2 features of EVOO polyphenolsmust be carried out in human pilot studies in the future, it is reasonable to suggest that parental and/or semi-synthetic derivatives of EVOO lignans and secoiridoids might represent previously unrecognized diet-based therapeutic strategies capable to successfully prevent and/or treat HER2-driven natural history of breast cancer disease.

This study reveals for the first time that all the major families of EVOO polyphenols (*i.e. *secoiridoids and lignans) represent previously unrecognized phytochemicals that significantly affect breast cancer cell proliferation and survival through a molecular mechanism involving, at least in part, a significant down-regulation of HER2 expression and activity (Figure 14). These findings, together with the fact that that humans have safely been ingesting significant amounts of lignans and secoiridoids as long as they have been consuming olives and EVOO, strongly suggest that the stereochemistry of these polyphenols might provide an excellent and safe platform for the design of new anti-breast cancer drugs. Although EVOO-rich has been linked with reduced breast cancer risk, and experimental studies begin to support the hypothesis of EVOO phenolics as inhibiting compounds of HER2-related breast cancer growth, important issues such as the accessibility of EVOO-derived secoiridoids and lignans to tumor tissues should be carefully addressed in animal models and human pilot studies. Only then an appropriate dietary intervention aimed to reproduce the prominent anti-oncogenic features of EVOO phytochemicals could be viewed as a new molecular approach in the management of HER2-positive breast cancer disease.

## Abbreviations

ATP: Adenosine triphosphate; ATCC: American Type Culture Collection; EVOO: Extra Virgin Olive Oil; CE-MS: Capillary Electrophoresis coupled to Mass Spectrometry; CE-ESI-MS: Capillary Electrophoresis-Electrospray Ionization-Mass Spectrometry; DAOA: Deacetoxyoleuropein aglycone; DMBA: Dimethylbenz(a)anthracene; ELISA: Enzyme-Linked ImmunoSorbent Assay; EGFR: Epidermal Growth Factor Receptor; FBS: Fetal Bovine Serum; HER1: Human Epidermal growth factor Receptor 1; HER2: Human Epidermal growth factor Receptor 2; HPLC: High-Performance Liquid Chromatography; IMEM: Iscove's modified Eagle's medium; MTT: 3-4, 5-dimethylthiazol-2-yl-2, 5-diphenyl-tetrazolium bromide; MUFA: Monounsaturated Fatty Acid; NAC: 6-hydroxy-N-acetylcysteine; OA: Oleic Acid; OD: Optical Density; OO: Olive Oil; PEA3: Polyomavirus enhancer activator protein 3; siRNA: small interfering RNA; TOF: Time of Flight.

## Competing interests

The authors declare that they have no competing interests.

## Authors' contributions

JAM, AVM and COF performed cell viability, apoptosis, ELISAs and drug treatments. JAM, AVM and ASC were responsible for data analysis. ACP and RGV performed semi-preparative reverse-phase HPLC for isolation of EVOO polyphenols and coordinated the chemical (ACP, RGV, AGF, ASC) and biological (JAM, AVM, COF) study groups. JAM, AFG and ASC participated in the design and coordination of the study design. JAM, AFG and ASC conceived the study, participated in its design, coordination and in the draft of the manuscript. All authors read and approved the final version of the manuscript.

## Pre-publication history

The pre-publication history for this paper can be accessed here:



## Supplementary Material

Additional file 1 **Supplementary materials.** Click here for file
