# Supplementary material for: tabAnti-HER2 (erbB-2) oncogene effects of phenolic compounds directly isolated from commercial Extra-Virgin Olive Oil (EVOO)
Source: BMC Cancer. 2008 Dec 18;8:377. doi: 10.1186/1471-2407-8-377 (PMC2626601; doi:10.1186/1471-2407-8-377)
Supplement: Additional file 1 — Supplementary materials. [file 1471-2407-8-377-S1.doc]

**Electronic Supplementary Material**

**Table I. Retention times, absorption maxima, and the mass spectrometry data using accurate mass for the eight isolated EVOO phenolic fractions**

| *Main isolated*  *EVOO polyphenols* | **UV** | | **TOF** | | | |
| --- | --- | --- | --- | --- | --- | --- |
|  | *t_ret_* | *λ_max_* | *m/z experimental* | *Formula (GMF)* | *Error (ppm)* | *Sigma* |
| **Hydroxytirosol** | 11.02 | 200/218/280 | 153.0555 | C_8_H_9_O_3_ | 1.242 | 0.0107 |
| **Tyrosol** | 16.20 | 196/218/276 | 137.0609 | C_8_H_9_O_2_ | -0.362 | 0.0048 |
| **Elenolic acid** | 36.40 | 200/218/240 | 241.0727 | C_11_H_13_O_6_ | -3.992 | 0.0210 |
| **DAOA** | 43.21 | 202/220/283 | 319.1198 | C_17_H_19_O_6_ | -3.287 | 0.0563 |
| **(+)-Pinoresinol** | 48.74 | 232/279/350 | 357.1336 | C_20_H_21_O_6_ | 2.024 | 0.0073 |
| **1-(+)-Acetoxypinoresinol** | 49.77 | 204/232/280 | 415.1386 | C_22_H_23_O_8_ | 2.979 | 0.0068 |
| **Oleuropein aglycone** | 56.48 | 234/279 | 377.1261 | C_19_H_21_O_8_ | -4.937 | 0.0144 |
| **Ligstroside aglycone** | 62.31 | 240 | 361.1283 | C_19_H_21_O_7_ | 2.704 | 0.0256 |

**Electronic Supplementary Material**

**Table II**. **Analytical parameters of the HPLC-MS method**

| *EVOO phenolic* | **Detection limit (μg/ml)** | **Calibration interval (μg/ml)** | **Calibration curve** | **r^2^** |
| --- | --- | --- | --- | --- |
| **Hydroxytyrosol** | 0.060 | QL-100 | y = 1312837,35x + 2888083,52 | 0,9881 |
| **Tyrosol** | 0.094 | QL-100 | y = 1069088,26x + 576012,73 | 0,9831 |
| **Elenolic Acid** | 1.850 | QL-200 | y = 176399,71 x + 471557,17 | 0,9915 |
| **DAOA** | 4.987 | QL-500 | y = 41290,45x + 123000,76 | 0,9391 |
| **(+)-Pinoresinol** | 0.652 | QL-200 | y = 23156,64x + 360293,33 | 0,9381 |
| **1-(+)-Acetoxypinoresinol** | 0.345 | QL-200 | y = 55426,71 x + 765902,97 | 0,9371 |
| **Oleuropein aglycone** | 0.556 | QL-200 | y = 136488,21x + 2216015,00 | 0,9391 |
| **Ligstroside aglycone** | 0.294 | QL-200 | y = 188216,09x + 2113758,02 | 0,9513 |

**Electronic Supplementary Material**

**Figure I**

**
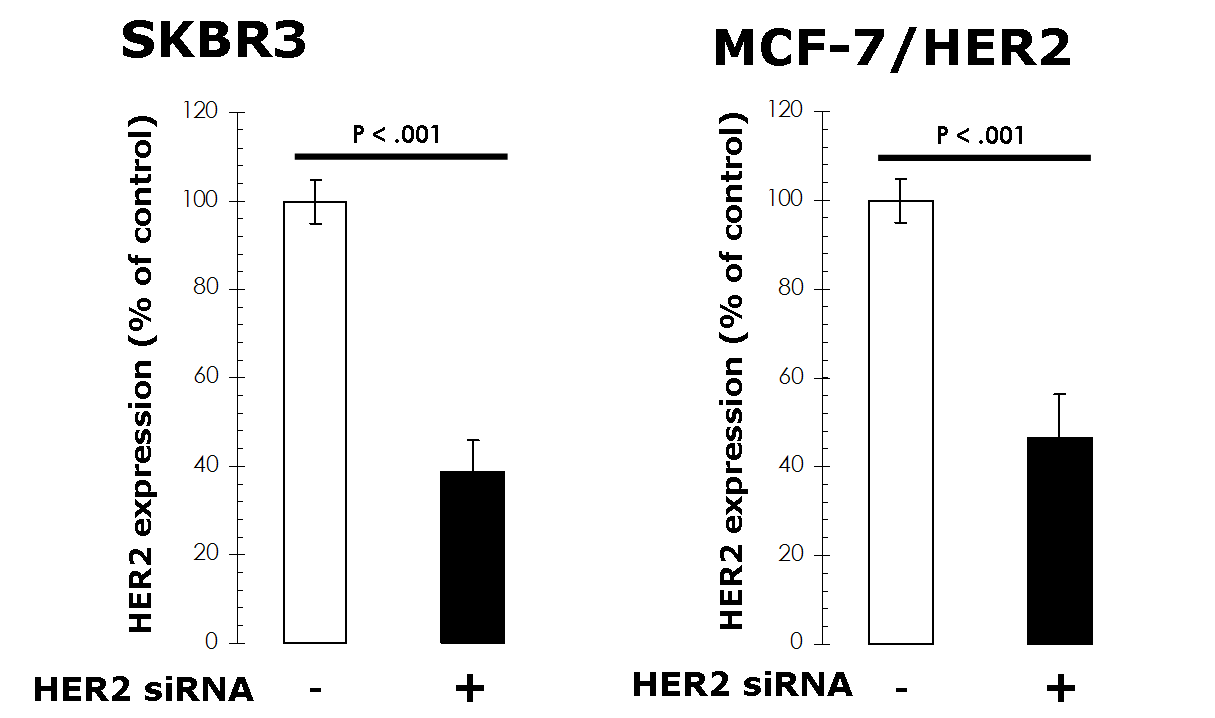
**

HER2 protein expression in whole cell lysates from SKBR3 and MCF-7/HER2 cells harvested 72 hr after the introduction of siRNA to HER2 or control siRNA (80 pmols/well) was quantified using the Oncogene Science HER2 microtiter ELISA according to the manufacturer’s instructions. HER2 expression in whole cell lysates transfected with control siRNA was set as 100%.

**Electronic Supplementary Material**

**Figure II**

**
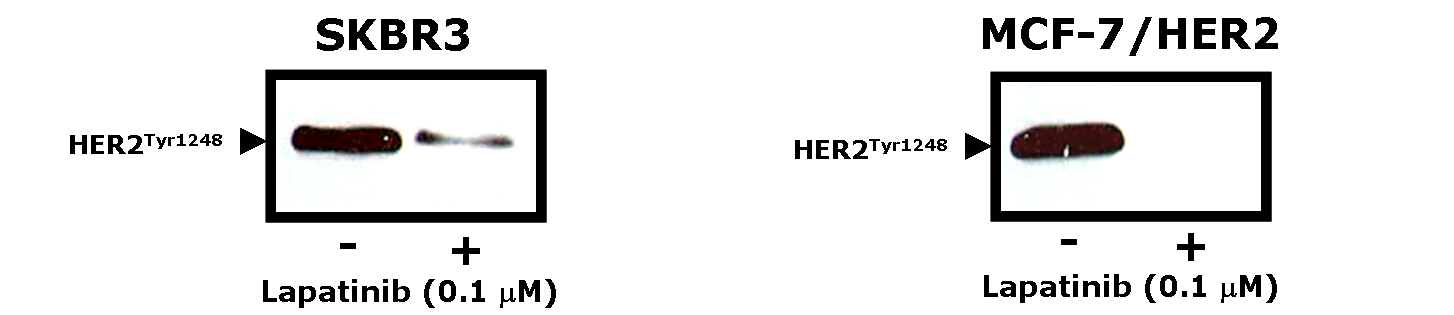
**

Overnight serum-starved SKBR3 (*left*) and MCF-7/HER2 (*right*) cells were treated with 0.1 μM lapatinib for 24 hr and tested for p185^HER2^ autophosphorylation at Tyr1248 using immunoblotting procedures as described in “Materials and methods”.

**Electronic Supplementary Material**

**Figure III**

**
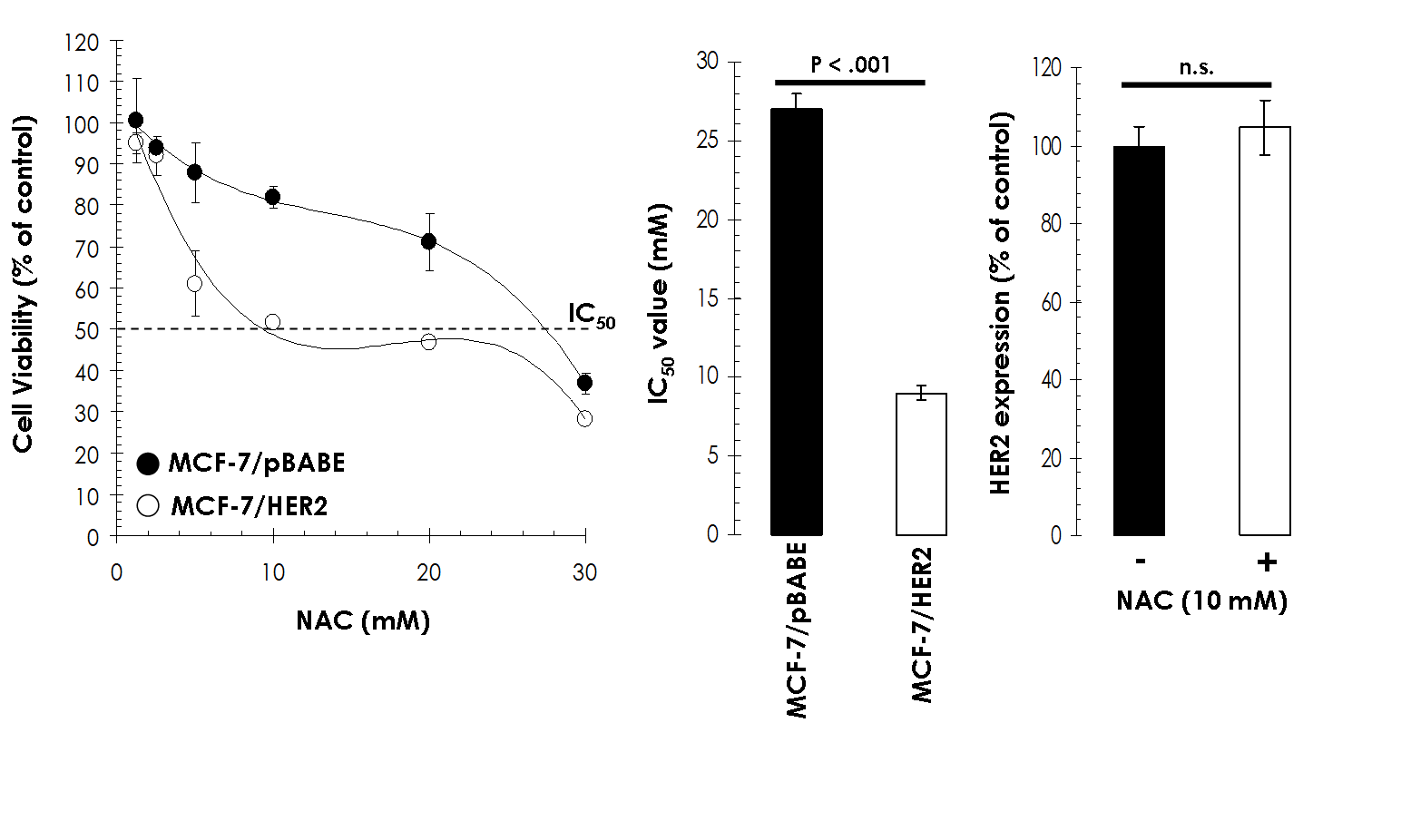
**

The metabolic status of NAC-treated MCF-7/HER2 and MCF-7/pBABE matched control cells was evaluated using a MTT-based cell viability assay and constructing dose-response curves as described in “Materials and methods”. Concentrations producing the IC_50_ value (the concentration of NAC needed to reduce cell viability by 50% relative to untreated control cells) were calculated by interpolation. Values are means (in mM) and 95% confidence intervals (95% CI) of three independent experiments made in triplicate. HER2 protein expression in whole cell lysates from MCF-7/HER2 cells harvested 48 hr after NAC treatment was quantified using the Oncogene Science HER2 microtiter ELISA according to the manufacturer’s instructions. HER2 expression in whole cell lysates transfected with control siRNA was set as 100%.

**Electronic Supplementary Material**

**Figure IV**

**
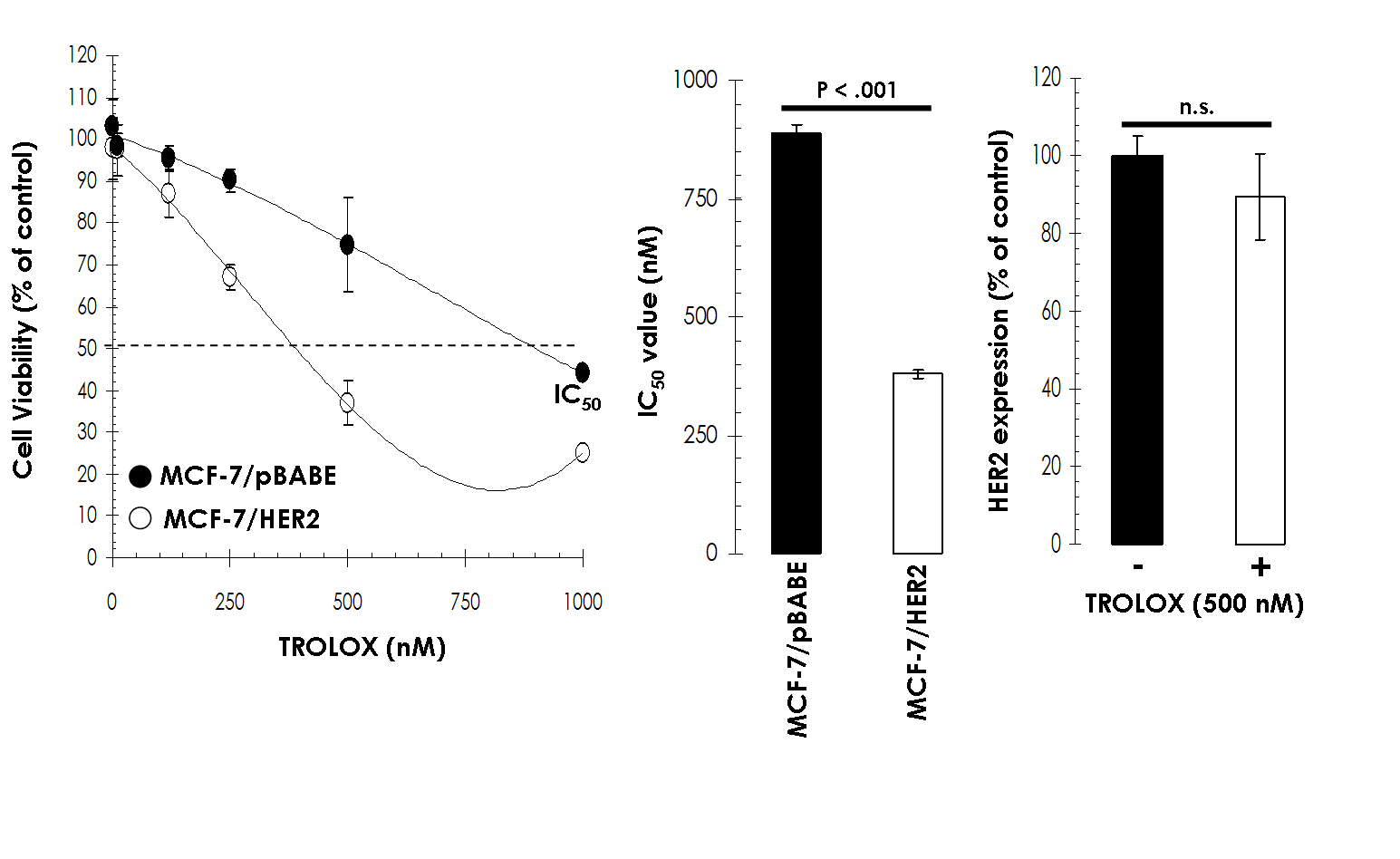
**

The metabolic status of Trolox-treated MCF-7/HER2 and MCF-7/pBABE matched control cells was evaluated using a MTT-based cell viability assay and constructing dose-response curves as described in “Materials and methods”. Concentrations producing the IC_50_ value (the concentration of Trolox needed to reduce cell viability by 50% relative to untreated control cells) were calculated by interpolation. Values are means (in nM) and 95% confidence intervals (95% CI) of three independent experiments made in triplicate. HER2 protein expression in whole cell lysates from MCF-7/HER2 cells harvested 48 hr after Trolox treatment was quantified using the Oncogene Science HER2 microtiter ELISA according to the manufacturer’s instructions. HER2 expression in whole cell lysates transfected with control siRNA was set as 100%.
